# Assessment of Dysfunctional Movements and Asymmetries in Children and Adolescents Using the Functional Movement Screen—A Narrative Review

**DOI:** 10.3390/ijerph182312501

**Published:** 2021-11-27

**Authors:** Pat R. Vehrs, Martina Uvacsek, Aaron W. Johnson

**Affiliations:** 1 Department of Exercise Sciences, Brigham Young University, Provo, UT 84602, USA; wayne_johnson@byu.edu; 2 Department of Health Sciences and Sport Medicine, University of Physical Education, 1123 Budapest, Hungary; uvacsek.martina@tf.hu

**Keywords:** motor competence, physical literacy, physical activity, physical fitness

## Abstract

The Functional Movement Screen (FMS) is a screening tool that identifies dysfunctional movements in seven test items requiring an interplay of cognitive, perceptual, proprioceptive, and motor functions that involve muscular strength/endurance, flexibility, mobility, coordination, and balance. The results of the FMS include an overall composite score, scores on the individual test items, and identification of compensatory movement patterns and left-right asymmetries on 5 bilateral test items. Although there is a plethora of literature on the use of the FMS in adults, there is a growing body of evidence indicating its use in children. The available research in children involves school children and young athletes in at least 20 different sports in over 20 countries and comparisons between pre- and post-pubescent children, and normal weight, overweight, and obese children. Studies that include measures of adiposity and physical activity levels, or report prevalence of asymmetries and dysfunctional movement patterns are not well represented in the children’s literature. The purpose of this paper is to synthesize the currently available literature in children and suggest potential uses of the FMS by coaches, physical educators, and other health/fitness professionals, appropriate interpretation of results, and future research in children.

## 1. Introduction

The most recent (2018) Physical Activity Guidelines for Americans recommends that in addition to moving more and sitting less, adults should engage in at least 150–300 min of moderate-intensity or 75–150 min of vigorous-intensity aerobic physical activity (or an equivalent combination of the two) per week [1,2]. Adults should also engage in muscle-strengthening activities that involve all the major muscle groups on at least two days per week [1,2]. The American College of Sports Medicine (ACSM) also recommends that older adults include neuromotor exercise training at least 2–3 days per week [3]. This type of training involving motor skills (e.g., balance, agility, coordination), proprioceptive exercise training, and multifaceted activities (e.g., yoga and tai ji) that improves and maintains physical function and reduces the risk of falls [3]. Although the recommendation for neuromotor exercise training is specifically directed to older adults, younger adults may also benefit from this type of training. In fact, in recent years, there has been a growing interest in neuromotor exercise training (aka functional exercise training) that emphasizes movement patterns with a focus on balance, coordination, stability, core stability, and proprioception training [4,5]. Functional movement has been defined as the ability to produce, and maintain a balance between mobility and stability along the kinetic chain, while performing fundamental movements with accuracy and efficiency [6]. A functional limitation is defined as a limitation in performance at the level of the whole person [7]. Functional movements are a complex interplay of cognitive, perceptual, proprioceptive, and motor functions that involve muscular strength and endurance, flexibility, coordination, and balance [6].

Declining levels of physical activity and increasing levels of overweight and obesity, and the subsequent negative impact on health outcomes in children and adolescents (hereafter “youth”) in the last four decades is concerning. Obesity occurs gradually, sometimes over several years. The progression toward obesity can include musculoskeletal pain, reduced physical function, impaired motor skills, and limitations in the ability to participate in regular physical activities [8]. The concerns for the increased prevalence of childhood obesity has prompted national and international organizations and governing bodies to develop physical activity guidelines for children and adolescents. The recent Physical Activity Guidelines for Americans recommends that children and adolescents 6 through 17 years of age do at least 60 min of moderate-to-vigorous physical activity every day [1,2]. Most of the 60 min of daily physical activity should be moderate-to-vigorous intensity aerobic activity but also includes muscle and bone strengthening activities [1,2]. A recent review revealed over 50 national or international physical activity guidelines for children that vary in the targeted age group and the recommended type, intensity, duration, frequency, and total amount of physical activity [9]. Physical activity guidelines for children encourage less sedentary and screen time, more physical activity throughout the day, and participation in a wide variety of age and developmentally appropriate structured and unstructured physical activities as part of play, recreation, and sports.

Although participation in various types of structured and unstructured activities can contribute to the neuromotor development of children, there does not appear to be specific recommendations for neuromuscular exercise training for youth [9] as there are for adults [3]. Nevertheless, the development of “motor competency” (mastery of movement patterns with coordination and control) is a growing emphasis in the development of physical literacy in youth [10,11,12,13]. Fundamental movement patterns that develop during early childhood are the building blocks to specialized movement patterns and participation in physical activity [13,14,15]. Dysfunctional and compensatory movement patterns develop when limitations in mobility, stability, or neuromuscular (motor) control prevent the practice of proper movement patterns [16]. As a child gets older, dysfunctional basic movement patterns integrate into games, sports, and recreational activities. 

A position statement by the National Strength and Conditioning Association (NSCA) states that functional movement is essential for the holistic development of the child and that children should participate in appropriate exercise programs that promote physical development and enhance fitness behaviors that are retained later in life [17]. Because an adequate level of motor competency is the foundation for a physically active lifestyle [12], there may be a level of movement competency which is predictive of future participation in physical activities [10,18,19,20]. In that light, it is appropriate to present some aspects of physical activity for children in the context of their future health and fitness as adults [15]. An age-appropriate emphasis on neuromotor exercise training (aka functional exercise training) and functional fitness in children and adolescents is worthy or exploration.

The purpose of this paper is to synthesize the currently available literature in children and suggest potential uses of the Functional Movement Screen (FMS) as a tool to assess quality of movement patters by coaches, physical educators, and other health/fitness professionals. We will also suggest appropriate interpretation of FMS assessments and future research in children.

## 2. Assessment of Physical Fitness

The physical and mental health benefits of regular physical activity are well documented [1,2,3] and continue to expand. Nevertheless, the primary purpose of an exercise program is to maintain and/or improve one or more of the components of physical fitness (e.g., cardiovascular fitness, muscle strength and endurance, flexibility, agility, balance, etc.). The assessment of physical fitness in youth in the United States has its roots in physical education. Over the years, social and political circumstances have influenced the training and assessment of physical fitness [21]. For example, prior to World War I, assessments included measures of motor ability. During and after World War I, there was an increased emphasis on physical education, physical fitness, and preparation for war. The interest in physical fitness testing faded during the Great Depression. World War II rekindled the military, governmental, and social interest in the training and assessment of physical fitness. In response to reports that American youth were less fit than their European counterparts, there was a rising concern for military preparedness, from which arose the President’s Council on Youth Fitness. During this same time, organizations such as the American Medical Association (AMA) and the American College of Sports Medicine (ACSM) advocated improvement in the physical fitness of the nation. The standardized assessment of physical fitness of youth in public schools in the Unites States began in the mid- to late 1950s. The battery of fitness tests included “health-related” assessments of muscular strength and endurance and cardiovascular fitness, as well as “skill-related” test items such as the softball throw and the 50-yard dash, which were thought to be related to athletic ability and military preparedness. The emphasis of youth fitness initiated by President Eisenhower in the 1950s continued with the advocacy for youth fitness by President Kennedy in the early 1960s.

The assessment of physical fitness of youth in a school setting is an educational process [22] that can be an important part of developing what has been termed, “physical literacy” [23] and motor competency. The Institute of Medicine (IOM) suggests that test items to assess physical fitness in children can be considered supplemental educational tools [22]. If used correctly, the assessment of physical fitness can be an essential component in the process of educating youth about and promoting and encouraging change in physical activity behaviors. The selection of tests used to assess physical fitness of youth must consider the overall efficacy of administering the tests as well as the educational value of the test [22]. The standardized assessment of physical fitness of youth currently used in public schools in the United States evaluates the five components of health-related physical fitness: muscle strength and endurance, flexibility, body composition, and cardiovascular fitness [22,24,25]. The most common tests used to assess muscle strength and endurance in children include the flexed arm hang, pull-ups, push-ups, curl-ups, and the trunk lift [25]. A call for additional research suggested that test items used to assess physical fitness of youth need to measure a broad range of fitness as well as dysfunctional movements [25]. Although in clinical practice, movement quality is regularly examined in children suspected of having motor dysfunctions [26,27], there are no specific guidelines for the assessment of movement quality in non-clinical and non-research settings by physical educators, fitness instructors, and coaches. 

## 3. Assessments of Movement in Children

A plethora of literature is available on the topic of fundamental movement skills (also annotated as “FMS” but not to be confused with the Functional Movement Screen) in children. Fundamental movement skills consist of locomotor skills that are used to move the body through space (e.g., walking, skipping, running, hopping, jumping); object control skills that manipulate an object (e.g., throwing, kicking); and stability skills (e.g., balancing, twisting) [28,29]. Age-appropriate fundamental movement skills are taught and assessed in school physical education programs using theories of motor development [30]. Children are thought to naturally develop rudimentary forms of fundamental movement skills which are then further developed with instruction and practice [14]. The fundamental movement skills are subsequently refined into more complex and sport-specific skills [14,28]. Mastery of fundamental movement skills is assumed in order for the child to participate (or be willing to participate) in and enjoy organized or informal activities [28]. The assessment of fundamental movement skills has previously been reviewed [14,28,29,31]. The observational assessment of fundamental movement skills should not be confused with the objective assessment of functional movements by the FMS that scores the quality of the movement and identifies dysfunctional and asymmetrical movement patterns. Recent research indicates that fundamental movement skills is relatively independent of scores on the FMS [32].

## 4. The Functional Movement Screen

Movement assessments should consider limb asymmetries, limitations in range of motion, proprioceptive deficits, posture, and pain [33]. The test items included in the FMS assess these parameters [34,35]. The FMS is a screening tool used to evaluate performance on seven different fundamental movement patterns. The movement patterns allow the observation of deficiencies in functional movement, neuromuscular control, balance, stability, and mobility [34,36]. The FMS identifies movement asymmetries and dysfunctional movement patterns that rely on compensatory movements. Deficiencies in functional movement may adversely affect physical performance and increase the risk of injury. Functional movement deficiencies may decrease participation in physical education, physical activities, recreation, and sports. 

The FMS includes seven fundamental movement patterns (test items): deep squat, hurdle step, in-line lunge, shoulder mobility test, active straight-leg raise, trunk stability push-up, and rotary stability test. The test items are assessed using standardized criteria [34]. Of these seven test items, five are bilateral tests performed on both sides of the body: hurdle step, in-line lunge, shoulder mobility test, active straight-leg raise, and rotary stability test. The five bilateral tests are used to assess the presence of asymmetries. The FMS test items permit the observation of mobility, stability, balance, and functional and dysfunctional movement patterns [34,36]. 

A detailed description of each of the FMS test items and their scoring criteria are described elsewhere [34,37,38]. Each test is performed three times. Tests that include bilateral movements (hurdle step, in-line lunge, shoulder mobility test, active straight leg raise, rotary stability test) are performed three times on each side of the body and scored independently. Each of the FMS test items are scored using a four-point system. A score of “3” indicates that the movement pattern was performed correctly; a score of “2” indicates that compensatory movements were involved in the movement; a score of “1” indicates that the movement was incomplete; and a score of “0” indicates that the individual reported feeling pain during the movement. A score of 1 represents a dysfunctional movement pattern. In the five bilateral tests, scores for both sides are recorded to note any imbalances and the lowest score recorded for either the left or right side is recorded as the score for the test item. The scores for all seven tests are summed to calculate a composite, or total score (maximal score of 21 possible points).

## 5. Definitions

*Motor Competency*. The degree of skilled performance in a wide range of motor tasks as well as the movement quality, coordination and control underlying a particular motor outcome [39].

*Fundamental Movement Pattern*. A basic movement that represents the foundation for the execution of more complex motor tasks; utilized to simultaneously assess range of motion, mobility, proprioception, motor control, stability, coordination, flexibility, and balance [34,35,37].

*Fundamental Movement Skills.* Locomotor skills (e.g., walking, skipping, running, hopping, jumping) that move the body through space; object control skills (e.g., throwing, kicking) that manipulate an object; and stability skills (e.g., balancing, twisting).

*Functional Movement.* A functional movement implies an optimal range of motion (flexibility of the soft tissue and mobility of the joints), balance, and neuromotor postural control of the body regions involved in the specific movement [34,35,40,41].

*Functional Movement Screen*. A screening tool used to evaluate performance on seven different fundamental movement patterns; observe deficiencies in functional movement, neuromuscular control, balance, stability, and mobility; and identify dysfunctional movements and asymmetries. 

*Dysfunctional Movement*. Suboptimal movement quality characterized by compensatory movement along the kinetic chain, observed asymmetry, and loss of range of motion, balance, posture, and motor control of the specific movement [34,35,40,41].

*Asymmetry.* A lack of equivalence between the left and right sides of the body in performing any of the five bilateral FMS test items: hurdle step, in-line lunge, shoulder mobility test, active straight-leg raise, and rotary stability test.

*FMS 4*. A partial FMS score based on four test items: deep squat, hurdle step, shoulder mobility, and active straight leg raise. The FMS4 is used in some of the studies involving obese children based on reports that obese children have difficulty performing the other 3 tests: in-line lunge, rotary stability, and trunk stability pushup.

*Movement score*. A sub-category FMS score used in some of the literature calculated by summing the scores on the deep squat, hurdle step, and in-line lunge.

*Mobility score*. A sub-category FMS score used in some of the literature calculated as the sum of the shoulder mobility and active straight leg raise test scores.

*Stabilization score***.** A sub-category FMS score used in some of the literature calculated by summing the scores on the trunk stability pushup and the rotary stability.

## 6. The Use of the Functional Movement Screen in Youth

Although there is a plethora of research on the use of the FMS in adults, there is growing scientific evidence indicating the use of the FMS in youth. Studies to date have been performed in the United States, Canada [42,43], Hungary and Germany [44,45], Italy [46], Moldova [38], countries of the UK [47,48,49,50,51,52,53,54,55,56,57], Ireland [58,59], Poland [60,61,62,63,64], Slovakia [65], Iran [66,67,68], Turkey [69,70,71,72,73,74], Japan [75], China [76,77], Thailand [32], Taiwan [78,79,80], Korea [81], Indonesia [82], Croatia [40,41,83,84], Brazil [85], Portugal [86], Spain [87,88,89,90,91,92,93], and South Africa [94], in both boys and girls, in physical education classes, in at least 20 different sports, in various age categories, and in normal weight, overweight, and obese youth. No studies to date have indicated the youth cannot complete the FMS. 

As already noted, the FMS is designed to identify dysfunctional movements. Inter-limb coordination and neuromuscular and postural control are not fully mature by adolescence [95]. The development and repeated practice of dysfunctional movement patterns through childhood and adolescence can lead to negative health consequences, injuries, postural abnormalities, movement pathologies, and orthopedic abnormalities later in life [40,41,48,83]. The source of dysfunctional movements can be a lack of physical activity and/or repetitive use of compensatory movement pattern during a growth period [96]. Thus, early identification of dysfunctional movements in youth is essential to the training and sustaining of functional movements through childhood and adolescence. Functional movements become the pillars for more complex movements [34,35,41].

### 6.1. Reliability of the Functional Movement Screen

The FMS is a screening tool designed to identify dysfunctional movements and asymmetries during standardized functional movement patterns. To be a valid assessment, the FMS must also be reliable within and between administrators (e.g., clinicians, coaches, physiotherapists). Reliable use of the FMS can help identify limitations, asymmetries, and dysfunctional movements. Improvements in movement quality due to an intervention would be more likely due to the intervention itself rather than an unreliable assessment.

Several studies have evaluated the intra- and inter-rater reliability of the FMS. For example, physiotherapists with FMS training certification administered the FMS to young elite ice hockey players [42]. Two physiotherapists evaluated each subject while performing each FMS test item. The two physiotherapists scored each subject performing each FMS test independently of the other to determine inter-rater reliability. Two other physiotherapists watched front- and side-view video recordings of subjects performing each of the FMS test items. Each of the physiotherapists evaluated the same video recording twice to determine intra-rater reliability. High intra-rater (ICC = 0.96) and inter-rater (ICC = 0.96) reliabilities demonstrate that the FMS is a reliable assessment tool. 

A meta-analysis of seven papers: 6 evaluating intra-rater reliability and 6 evaluating intra-rater reliability, indicated good inter-rater reliability (ICC = 0.843; *p* = 0.001) and good inter-rater reliability (ICC = 0.869; *p* = 0.001). Coaches, clinicians, and physiotherapists could integrate the FMS into their team practices or clinical exams to recommend appropriate interventions that focus on movement pattern deficits.

### 6.2. Grouping Individual FMS Test Items into Sub-Scores

In recent years, a few studies have grouped the individual FMS test items into categories and report sub-scores. A “movement” score is calculated by summing the scores on the deep squat, hurdle step, and in-line lunge. A “mobility” score is calculated as the sum of the shoulder mobility and active straight leg raise test scores. A “stabilization score” is calculated by summing the scores on the trunk stability pushup and the rotary stability.

A recent study found no significant differences (*p* > 0.05) in the total FMS score, movement score, mobility score, or stabilization score between young male soccer players who either lean (less than 6% body fat) or normal (6–18% body fat) [97]. After following a corrective exercise program involving the use of medicine balls, elastic bands, foam rollers, self- and partner stretching, and strength and stability exercise, there was an increase in the total FMS score as well as the movement, mobility, and stabilization sub scores in the training group but not the control group [46].

### 6.3. Effects of Prior Knowledge on FMS Scores

An important question to ask about the FMS is if prior knowledge of the grading criteria improves performance on the FMS. In other words, if participants are given information about the movement patterns required to achieve the perfect score (score = 3), would there be an improvement in scores? We found one study that evaluated the effects of prior knowledge of the grading criteria on the performance on the FMS in young soccer players [47]. In this study, youth performed the FMS twice. For the first trial, none of the subjects received information about the grading criteria. During the second trial, half of the youth received information about the grading criteria. The group that received no information about the grading criteria for either trial had identical scores on both trials. The group that received information about the grading criteria during the second trial improved their total FMS score by 2 points. 

### 6.4. Using a 4-Item vs. 7-Item FMS

Based on previous reports that the in-line lunge, trunk stability push-up, and rotary stability tests were difficult for overweight and obese youth to perform [49] some studies have used the FMS4 to evaluate movement competency in overweight and obese youth [87,91,92,98]. The FMS4 includes the deep squat, hurdle step, shoulder mobility, and active straight leg raise. The FMS4 is not part of the original FMS [34,35]. The individual test items are scored the same (0, 1, 2, 3) and the total FMS4 score ranges from 0–12.

Compared to overweight children, obese children scored significantly lower on the FMS4 (8.2 vs. 6.4, respectively) and the deep squat, hurdle step, and shoulder mobility individual test items [91]. Likewise, in a study of 62 overweight and obese children, the obese scored significantly lower than overweight children on the FMS4 (5.8 vs. 8.3, respectively) [98]. The FMS4 has also been used in training studies involving overweight and obese youth. In a 13-week training study, overweight and obese boys and girls improved their performance on the deep squat and active straight leg raise (FMS4 score not reported) [92]. Likewise, following 16 weeks of physical conditioning, overweight and obese children increased their FMS4 score from 5.35 to 6.94, and their FMS7 score from 8.76 to 10.7 [87]. Some of the implications of performance on the FMS in overweight and obese children is discussed in Section 6.8 below.

### 6.5. The FMS in Youth Sports and Risk of Injuries

Many of the studies have been descriptive in nature, evaluating movement competencies of young athletes in soccer [46,47,50,51,53,56,66,69,86,97,99,100], Australian football [101], football [99,102], basketball [70], baseball [80], floorball [64], swimming [54,103], ice hockey [42,43,63], karate [62], judo [88], tennis [73,74], cross country and volleyball [61,99], cricket [94], handball [79], lacrosse [55,102], Wushu (Taiji, Changquan, Nanquan) [77], and surfing [86].

In 1980, Seefeldt [104] introduced the concept of a “proficiency barrier” which can be viewed as a threshold, above which a child is able to successfully transition movement skills into more complex movement patterns and be more likely to engage in various forms of physical activity since they have developed necessary movement competencies [39,55]. A child with motor competence below the proficiency barrier is less likely to engage in certain activities since he/she would not have the required skills, confidence, and motivation to successfully participate [39]. 

In adults, the concept of a proficiency barrier has been applied to the ability of the FMS to identify individuals at increased risk of injury. As the FMS screens for dysfunctional movements and asymmetries during functional movement patterns, it may be able to identify individuals who are an increased risk of injury during physical activity, recreation, and sports participation. The most commonly cited proficiency barrier, or threshold, in adult athletes is based on the seminal study by Keisel et al. [37] who reported a threshold total FMS score of 14 below which there is an eleven-fold increased risk of injury.

Studies evaluating the relationship between the FMS score and injury risk in children have been done in a variety of sports, including cricket [94], baseball [80], basketball and handball [79], floorball [64], soccer [51,55,71,72,99,100,105,106], football [99,102], lacrosse [55,102], and volleyball [55], Wushu (Taiji, Changquan, Nanquan) [77], and basketball and handball [79], hockey [43], swimming, cross country, and tennis [99], and Australian football [101]. The assumption is that higher levels of movement competency (as represented by higher FMS scores) provides some protection from injury. Contrary to the findings of a threshold FMS score that is predictive of injury rates in adults, the composite FMS score has consistently been found to be a poor predictor of injury rates in young athletes in a variety of sports [43,51,72,79,80,94,99,100,101,102,107].

Some of the literature reporting on the association between the FMS score and injuries suggest that the threshold for increased risk of injury in youth is higher than that reported for adults (FMS ≤ 14). For example, young floorball athletes who sustained injuries had an average FMS score of 17 [64] and a FMS score less than 16 (sensitivity = 80%; specificity = 56%) was related to an increased occurrence of injury in Wushu athletes [77]. In a study of 136 athletes (63 boys; 73 girls) involved in various sports (football, soccer, volleyball, lacrosse), FMS scores <15 increased the odds of injury but after adjusting for sport, there was no FMS score relating to increased odds of injury [55].

Two studies have evaluated the performance on the FMS in soccer players who were previously injured [71,105]. Both studies report that there were no differences in the FMS score between previously injured and uninjured athletes. In addition, the presence of a previous injury did not affect the FMS subgroup scores, scores on individual test items, or the distribution of asymmetries [71,105]. Nevertheless, data from a recent study of elite male soccer players indicates that players with a history of hip and back injuries are at greater risk of sustaining a future soccer-related musculoskeletal injury [106]. Intriguing data presented in this paper were that larger players and players with higher FMS scores were at increased risk of injury. For each 1-point increase in the total FMS score, there was nearly a 2-fold increase in the odds of sustaining a soccer injury. One possible explanation for this is that larger players and players with higher FMS scores had more play time and were thus more likely to be injured.

### 6.6. Use of the Functional Movement Screen in Schools

Two studies have infused the FMS in school physical educations programs. A recent study in Taiwan [78] used the FMS to assess the benefits of core conditioning as part of a warm-up routine in physical education classes on movement quality. Fifty-two children (28 girls; 24 boys) 10–11 years of age were assigned to one of two groups: one group performed a general warm-up routine while the second group performed 10 min of dynamic core exercises. Trunk muscular endurance, movement quality (as assessed by the FMS), and flexibility were assessed before and after a 6-week intervention (2 days/week). The data indicated that after 6 weeks, the FMS score significantly (*p* < 0.001) increased in both groups of children, but the children who performed dynamic core exercise had a greater increase in their total FMS score as well as measures of trunk muscular endurance and flexibility. The authors concluded that children can increase their core strength and endurance over time, enabling them to maintain postural control and reduce inefficient movement patterns. The greater improvement in all measured parameters in the dynamic core training group suggests that traditional warm-up activities, although beneficial, are suboptimal [78]. This conclusion concurs with another study that infused functional warm-up activities into physical education classes. Compared to children who performed traditional warm-up activities, children who performed functional warm-up activities over a 6-week period reduced the prevalence of dysfunctional movement scores (score = 1) and boys in particular also improved their total FMS score [16]. 

### 6.7. Sex and Age Differences in the Performance on the FMS

Performance on the FMS can include comparisons between boys and girls on the total FMS score, scores on individual test items, and prevalence of asymmetries and dysfunctional scores. Of the available studies, some include both boys and girls [32,40,41,44,54,55,57,58,59,62,63,76,77,78,81,82,83,84,86,87,88,89,91,92,93,103,108], some include only boys [43,46,47,50,51,53,56,61,64,66,68,69,70,71,72,73,74,75,80,85,90,94,97,100,101,102,105,106,109] and some studies do not report the sex of the participants at all [14,52,60,65,67,79,86]. Few, if any studies include only girls.

Of the studies that included both boys and girls, some studies have not reported sex differences in the FMS [57,77,78,82]. Other studies have reported no significant differences in the FMS scores between boys and girls [36,38,41,44,48,49,58,62,63,76,81,83,86,87,89,90,91,93,99,103,110]. Fewer studies have reported that girls perform better than boys [32,40,45,54,55,59,88,108] and even fewer studies report that boys perform better than girls [107,111].

A recent study [45] of 10 to 12-year-old girls and boys, girls (FMS = 14.1) had a significantly higher FMS score than boys (FMS = 12.9). This concurs with a previous study [48] in which 10 to 11-year-old girls (FMS = 14.5) had higher FMS scores than boys (FMS = 13.5). In an older group of youth, 10 to 17-year-old boys (FMS = 14.9) had higher FMS scores than girls (FMS = 14.1) [107] but the difference was less than one point. In a group of athletic 13 to 18-year-old boys and girls, boys (FMS = 15.3) had higher FMS values than (FMS = 13.8) [111]. In the study by Vehrs et al. [45] and Duncan et al. [48], non-athletic participants were 10 to 12 years of age whereas participants in other studies were either younger [38,49] or older [99,110]. Other studies include athletes [99,110,111]. 

In addition to sex differences in the total FMS score, there are also sex difference in how boys and girls perform on individual FMS test items. Generally, boys have more dysfunctional movements in movement patterns that challenge mobility and flexibility and girls underperform boys in movements that require upper body strength and core stabilization [40,45,81,88]. For example, in a group of 10 to 17 year old youth, boys performed better on the trunk stability push-up, in-line lungs, and rotary stability tests [107] and an older (14–18 years of age) group of boys performed better on the trunk stability push-up test and the inline lunge [111]. Girls often perform better on the deep squat and the active straight leg raise [45,88]. In a group of elite junior athletes 16 to 20 years of age, there were no sex differences in any of the individual FMS test items [44]. 

Generally, older children perform better on the FMS than younger children [50,110]. In a study of 11 to 15 year old soccer players, there was a non-significant upward trend in the total FMS score from the 11 year-olds (FMS = 15.7), 13 year-olds (FMS = 16) to the 15 year-olds (FMS = 17.2) [65]. This is in agreement with a recent study [45] in which there was a weak, but positive correlation between age and the FMS score in 10 to 12 year old girls (0.242) and boys (0.053). The weak correlation was likely due to the narrow age range of the participants. To the contrary, a previous study reported a weak negative correlation (−0.038) between age and the FMS score in 10 to 17 year-old youth [107].

Karuc et al. [40] recently presented three possible explanations for sex differences in performance on the FMS observed in some studies: physiological (differences in muscle performance), anatomical (differences in joint architecture and mobility), and sociocultural (differences in participation in activity and sport). Both extrinsic (age, sex, maturation level, and fitness level) and intrinsic factors (muscle activation, neuromuscular control, and core stability) also influence movement quality and therefore the FMS score [111]. These factors are highly dynamic and trainable and improve with normal growth and maturation [110,111]. Because age and sex differences in the FMS reflect differences in a number of factors–rather than just because the individual is older or younger or because the individual is a boy or a girl–age and sex difference must be interpreted with caution. 

### 6.8. Performance on the FMS in Normal Weight, Overweight, and Obese Children

One theory of motor development is that individual constraints (internal structural or functional constraints), task constraints (external constraints specific to the demands of the movement), and environmental constraints (external physical or cultural) interact during movement [112,113,114]. Individual functional constraints include the individual’s body structure (e.g., body size, body composition, somatotype) [113]. As the prevalence of obesity among children and adolescents persists, some of the attention focusing on the health consequences of obesity has shifted to evaluating the impact of obesity on physical functioning and disability. Several reviews have indicated that childhood obesity, much like obesity in adulthood, is associated with deficits in function and performance of motor tasks, musculoskeletal pain, injury, decrements in muscle strength, gait, and balance [115,116]. Excess body weight imposes functional limitations and overweight and obesity alters functional movements in children [117,118]. As noted above, some studies have used the FMS4 rather than the 7-item FMS to screen movement quality in overweight and obese youth. For example, obese children (FMS = 6.4) scored significantly lower than their overweight counterparts (FMS = 8.2) on the FMS4 and on the deeps squat, hurdle step, shoulder mobility individual test items [91]. 

Studies using the 7-item FMS have reported mixed results. In a group of 652 youth (321 girls; 331 boys), there were no differences in the FMS score between normal weight boys (FMS = 12.6) and girls (FMS = 12.8) but overweight/obese boys (FMS = 11.1) significantly (*p* = 0.001) underperformed overweight/obese girls (FMS = 12.6) [40]. When comparing the prevalence of dysfunctional scores, normal weight and overweight/obese girls had a similar number of dysfunctional scores in all individual FMS test items except for the shoulder mobility test, in which case overweight/obese girls had a greater prevalence of dysfunctional scores. To the contrary, overweight/obese boys had a greater prevalence of dysfunctional scores in five (deep squat, in-line lungs, shoulder mobility, trunk stability push-up, and rotary stability) of the seven test items. The results suggest that the association between adiposity and performance on the FMS is sex specific. A study evaluating the influence of body composition on FMS scores in young male soccer players [97] also reported that there were no significant differences in the FMS scores between “lean” (<6% body fat) athletes and “normal” (6%–18% body fat) athletes. A possible explanation for this finding is that there were no differences in percent body fat (5.15% vs. 8.20%) and BMI (21.34 kg/m^2^ vs. 22.04 kg/m^2^) between the lean and normal groups, respectively. 

A recent study [45] reported that underweight (FMS = 14.2) or normal weight (FMS = 13.9) children had significantly higher FMS scores than overweight (FMS = 12.1) or obese (FMS = 11.8) children. This study also reported that the majority of individual FMS test item scores and the total FMS score were negatively correlated to indicators of obesity such as BMI, BMI percentile rank, and percent body fat. Of all the measures of adiposity, percent body fat had the highest correlation with the total FMS score. The correlation between percent body fat and individual FMS test items varied between the test items and sex, suggesting that the influence of body composition on individual test items is sex specific [45]. The influence of adiposity on the performance of individual FMS test items may be related to the distribution of body fat and the strength, balance, or motor control requirements of the test item, or if the movement involves primarily the upper body or lower body. Other studies [48,49] also reported negative correlations between the FMS score and BMI in children and that underweight (FMS = 15.5) and normal weight (FMS = 14.7) children had significantly higher total FMS scores than overweight (FMS = 10.6) or obese (FMS = 9.0) children.

Somatotyping is method of classifying individuals by body type. The three main somatotypes are endomorph (relative fatness), mesomorph (relative musculoskeletal development), and ectomorph (proportional height and weight). Although it was not intended to review the literature of somatotyping of children and adolescents in this paper, it is important to note that links between somatotype and physical fitness in youth have been reported. For example, there is a positive association between ectomorphy and motor ability and negative associations between endomorphy and mesomorphy and motor ability [119]. To the best of our knowledge, there are no studies in youth than have specifically evaluated the relationship between somatotype and performance on the FMS.

### 6.9. Asymmetries and Dysfunctional Scores

The scoring system of the FMS allows for the identification of pain during movement (score = 0), dysfunctional movements (score = 1), and asymmetries (different scores on the left and right sides of the bilateral movements). Section 6 above discusses the importance of identifying dysfunctional movements in youth. Using the FMS to assess asymmetries is important because having asymmetrical movement patterns in two or more individual tests items increases the risk of injury [109]. Nevertheless, many studies that use the FMS to assess motor competency in youth do not report the prevalence of asymmetries in bilateral tests or dysfunctional scores [32,40,41,47,53,58,59,61,62,63,69,73,74,76,78,79,81,82,83,84,87,89,90,91,93,106,108].

We have found few studies that have reported that youth have pain while performing the FMS. For example, young ice hockey players reported pain during all 7 test items, the most prevalent (15/28 players) being the trunk stability pushup and the least (1/28 players) being the hurdle step and the rotary stability test [42]. The reporting of pain by youth while performing the FMS may be under-represented in the literature as most studies also fail to report the prevalence of individual test item scores (i.e., 0, 1, 2, 3), dysfunctional movements, and asymmetries.

From those studies that report asymmetries, a high prevalence of youth that have at least one asymmetry becomes apparent. In a study of 14 to 20 year old soccer players, the prevalence of asymmetries varied by age, but asymmetry was found in at least one bilateral test in 65% of the athletes [85]. Likewise, in a group of 13 to 19 year old male soccer players, 68% of the players had at least one asymmetry, with the greatest number of players having asymmetry in the shoulder mobility test [72]. Similarly, the shoulder mobility test had the greatest number of asymmetries in both boys and girls 16 to 17 years of age [40]. In the same study of 362 boys and 368 girls, 79% of boys and 76% of girls had at least 1 asymmetry and 27% of boys and 30% of girls had 2 asymmetries [40].

Vehrs et al. [45] reported that 55% of 10 to 12 year old boys and 68% of girls presented at least one asymmetry, which is similar to the 64% and 61% prevalence of at least one asymmetry in 8 to 11-year-old boys and girls, respectively [38]. Three studies that included 13 to 18-year-old male participants reported a 63% to 68% prevalence of at least one asymmetry [101,102,120].

In the Vehrs et al. study [45], the greatest number of asymmetries occurred on the shoulder mobility test for boys and the rotary stability test for girls. This is in agreement with two studies in boys that reported that the greatest number of asymmetries occurred in the shoulder mobility test [101,120]. Mitchell et al. reported that both boys and girls had the greatest number of asymmetries when performing the hurdle step [38]. 

A dysfunctional score (score = 1) on the FMS indicates that the participant was unable to complete the movement as instructed. In the Vehrs et al. study, 86 of the 94 children (92%) had a dysfunctional score on at least one FMS test item which is in agreement with the 93% of male cricket players [94] and 85% of 7th and 8th grade physical education students [16] previously reported. Fewer (68%) high school athletes have been reported to have dysfunctional scores [102]. The greatest number of dysfunctional scores in 10 to 12 year old boys and girls [45], high school swimmers [103], and swimmers [103] occurred in the trunk stability pushup test. Other studies reported the greatest number of dysfunctional scores on the deep squat [43,101,103], rotary stability [94], and the shoulder mobility [43] test items. 

### 6.10. Effects of Exercise Training on FMS Scores

Several studies have investigated the effects of a pre-exercise warm-up and exercise training on FMS scores in youth [46,52,66,70]. Following 8 weeks of core stabilization training, 12 to 14-year-old male basketball players increased their total FMS score from 13.5 to 15.2 [70]. In a study of high school baseball players, 12 weeks of FMS training 4 days/week resulted in an increase in the average total FMS score from 13.7 to 17.5 and a decrease in the number of athletes who had a total FMS score of <14 [75]. A study of the effects of 12 weeks of functional training on young (age 17) male competitive footballers (soccer) resulted in increases in scores on the deep squat, hurdle step, and in-line lunge [60]. In this study, not all seven test items of the FMS were performed. Overweight and obese children (boys and girls) participated in a 13-week (3×/wk) exercise program based on movement quality [92]. This study used only four of the FMS test items: deep squat, hurdle step, shoulder mobility, and active straight leg raise. Following 13 weeks of training, there was an increase in the scores for the deep squat and the active straight leg raise. In a group of 14–19 year old male footballers, 12 weeks (2×/wk) of training (4 weeks of mobility training; 4 weeks of mobility training; 4 weeks of integration exercise) resulted in an increase in the total FMS score and scores on the deep squat, hurdle step, in-line lunge, and trunk stability push-up test items [69]. There was also a decrease in time missed due to injury and increased play time in the training group. In a group of 14-year-old male volleyball players, 12 weeks (2×/wk) of stabilization training increased their total FMS score and scores on the deep squat, trunk stability push-up, and rotary stability [61]. Following 4 weeks (3×/wk) of total resistance exercise system (TRX) training, there was 3-point increase in the total FMS score in 12 to 16-year-old children (sex not reported) [67]. Training of 10 to 11-year-old school children (boys and girls) during their Physical Education class for 6 weeks (2×/wk) resulting in an increase in the total FMS score from 13 to 15 [78]. Incorporating the FIFA + 11 program (a comprehensive warm-up program involving running, strengthening, jumping, soccer movements, and balance exercises) for four weeks (3×/wk) into the weekly training of young soccer players resulted in an increase in the mean FMS score (12.4 to 14.4), a decrease in the number of players scoring less than 14 on the FMS, and a decrease in the number of players with asymmetries [66]. A recent study in male soccer players evaluated the effectiveness of a 20-week corrective exercise program on the total FMS score and the movement, mobility, and stability sub-scores [46]. The corrective exercise intervention significantly improved the total FMS score, movement sub score, and stability sub score and decreased the number of asymmetries and dysfunctional scores. The effects of 4 weeks of a FIFA + 11 program (a comprehensive warm-up program involving running, strengthening, jumping, sprinting, balance exercises, and soccer movements) was evaluated in junior soccer players [66]. Inclusion of the FIFA + 11 program in the training practice 3×/wk significantly increased the total FMS score from 12.4 to 14.4 and reduced the number of asymmetries [66]. Wright et al. [52] evaluated the effect of 4-weeks of functional movement training on FMS scores and other measures of physical fitness, such as the plank, side plank, sit-and-reach, and a cardiorespiratory fitness (VO_2max_). The emphasis of the training was movement quality with exercises such as crawling, lunging, squatting, prone plank, pushups, and jumping. Contrary to the results of other studies, the 4 weeks of training had only a trivial effect on the total FMS score.

Despite the importance of reporting the prevalence of asymmetries and dysfunctional scores (score = 1 on an individual test item) among youth, most training studies failed to report the prevalence of asymmetries and dysfunctional scores before and after training [61,69,70,75,78,92]. 

## 7. Synopsis and Conclusions

In recent years there has been a dramatic increase in the number of studies that have used the FMS to screen for dysfunctional movements in children and adolescents. Nevertheless, some studies are more informative than others since many lack complete disclosure of data, such as sex of the participants, scores on individual test items, prevalence of dysfunctional scores and asymmetries, sex differences in scores, and measures of adiposity. It should be appreciated that studies that have data on the total FMS scores, also have data on individual test item scores, asymmetries, dysfunctional scores, and sex differences. The advancement of our understanding of the use of the FMS in youth will require that future studies report all of the data. 

*Reliability.* Generally, the FMS is a reliable tool that can be used to assess quality of movement in children and adolescents in a variety of settings. Reliability of the FMS depends, in part on the training and experience of the test administrator. When using the FMS in physical education classes or sports teams, the reliability of assessing movement quality should first be determined between multiple physical educators and coaching staff.

*FMS sub-scores*. Performance on the FMS can be reported at a total score as well as movement, mobility, and stability sub-scores. The benefit of reporting sub-scores is that differences between boys and girls or differences between age groups may be apparent in sub-scores when there are no differences in the total FMS score. Use of sub-scores may also be the basis for targeting an exercise program on movement, mobility, or stability components or better describe the effectiveness of a training intervention on specific movement deficits.

*Prior knowledge of grading criteria*. Performance on the FMS is influenced by awareness of the grading criteria. This can infringe on the objectivity of the test as well as its reliability. If participants are informed of the grading criteria prior to performing the FMS, the FMS may not be suitable to evaluate the effects of training or other intervention programs. Thus, in the light of remaining as objective as possible during a screening, it is recommended that participants remain blind to the grading criteria. If multiple screenings will be performed, for example, before and after an intervention, it is recommended that participants be blind to the grading criteria during the entire intervention.

*Four-item* vs. *seven-item FMS*. A 4-item version of the FMS (FMS4) has been used to accommodate overweight and obese youth who have difficulty performing three of the individual test items. This may mask the overall motor competency of overweight and obese children. Using the FMS4 instead of all seven items of the FMS creates a bias in the assessment by including only the test items that are easier to perform. We recommend having youth perform all seven test items, reporting a total FMS score as well as scores on individual test items, sub-scores, dysfunctional scores, and asymmetries.

*Identifying risk of injury*. The consensus of the literature is that the total FMS score is not a good predictor of risk for injuries in youth. We do not recommend the use of a threshold FMS score established in adults (FMS = 14) to identify increased risk of injuries in youth. Some evidence suggests that higher thresholds exist in youth, but this requires well-structured studies to further evaluate the FMS as a tool to identify risk of injuries. Identifying an injury risk threshold is complicated by the many variables than contribute to injuries in youth. Rather than using the FMS to derive an overly emphasized single FMS score, it can be used to develop a profile of current or prospective athletes [53] that includes scores on individual test items, sub-scores, asymmetries, and dysfunctional scores that establish the basis of a training program to facilitate overall improvement.

*Use of the FMS in schools*. As public and private schools have an influence on large numbers of youth most days of the week, physical education classes, after school programs, and athletic programs are a reasonable venue to evaluate motor competency of children and implement appropriate interventions. School physical education programs may have the greatest potential impact since they are generally inclusive of all children. Since assessments in physical education programs are common, the FMS could be used as an additional educational tool to assess and inform children of aspects of physical activity that are not evaluated in traditional physical fitness tests. Like other assessments of physical fitness, functional movement screenings in some children (particularly those who are less active, overweight, or obese) may introduce shame, discouragement, and other perceptions that may be barriers to increased involvement in physical education, physical activity, recreation, and sports. Use of the FMS should be in the context of the overall educational process. The literature is clear that overweight and obese youth perform poorly on the FMS. As noted above, we recommend using the entire 7-item FMS rather than the FMS4 which may bias, or mask, the movement dysfunctions in overweight and obese youth. In youth, the results of a functional movement screening can help objectively identify dysfunctional movements and asymmetries that may help explain poor performance in other kinds of physical activity and be the basis for appropriate interventions that could change the trajectory of participation in physical activity, recreation, and sport during adolescence and into adulthood.

Reports about the use of the FMS in physical education programs in public schools is encouraging. Implementing a warm-up routine that includes various functional movements is a plausible alternative to the traditional warm-up routine in physical education classes [121]. Such a routine would include exercises that focus on developing mobility, stability, motor control, and integrating these characteristics into movement patterns [121]. 

*Sex and age differences in the FMS*. There is inconsistent evidence of sex differences in performance on the FMS in youth. A lack of sex differences would imply that a combined male/female mean FMS score would represent a normative value for the FMS, as previously suggested [107]. To the contrary, data from studies that report sex differences in the performance of the FMS implies the need for sex-specific norms. When sex differences do exist, they must be interpreted with caution. Various factors contribute to the development of movement quality (and performance on the FMS) during childhood. Motor competency requires muscle activation and muscle strength, proprioception, neuromuscular control, core stability, balance, coordination, flexibility and joint mobility. These factors are highly dynamic, so sex and age differences are likely to diminish as muscle strength, neuromuscular control, core stability, balance, coordination, flexibility and joint mobility improve with age, growth and maturation, and involvement is various forms of physical activity. The presence of sex differences in movement quality invites a closer examination of the underlying factors that contribute to these differences. If the differences are primarily sociocultural, rather than physiological or anatomical [40] then an evaluation of physical education programs and availability of physical activities may be warranted. Because of the dynamic nature of movement quality, the generalizability of a single normative FMS score during childhood is not appropriate. Instead, the FMS score, individual test item scores, asymmetries, and dysfunctional scores should be considered in developing an appropriate exercise program with the intention to improve motor competence.

*Dysfunctional scores and asymmetries*. There is a high prevalence of dysfunctional scores and asymmetries in youth that spans boys and girls and all ages. The differences in dysfunctional scores and asymmetries are likely related to the same variables that explain sex and age differences. The high prevalence of dysfunctional scores and asymmetries alone should drive changes in the current physical education, after school, and athletic programs that focus on the overall needs of the child or athlete. Failing to report the individual test item scores, asymmetries, and dysfunctional scores is a gross underutilization of the potential of the FMS. Having a better understanding of dysfunctional scores and asymmetries is more valuable than knowing the single total FMS score. Grouping individual test items into movement, stability, and mobility sub-scores may be useful in identifying general areas of concern and the development of an appropriate exercise program designed to improve motor competency.

*Training effects on the FMS*. The available reports that evaluate the influence of various warm-up activities or training routines are promising. To the best of our knowledge, all of the training studies reported some benefit to participants, which included an increase in the total FMS score or its movement, stability, or mobility sub-scores, or a decrease in the prevalence of dysfunctional scores and asymmetries. The outcomes likely vary with the selected exercises, duration of the intervention, and compliance with the exercise program. The available data suggest that improvements in motor competency, as evidenced by improvements in performance on the FMS, are possible in youth with relatively simple interventions. In addition, measurable improvements occur over relatively short periods of time. This is encouraging for those individuals who have motor dysfunctions and for physical education teachers and athletic coaches.

## 8. Directions for Future Research

Future studies should clearly be more transparent in the reporting of data, including information about the sex and age of the participants, measures of body size or body composition, prevalence of asymmetries and dysfunctional scores, and sex differences in the FMS score as well as individual test items. The relationship between adiposity and performance on the FMS (total score, dysfunctional scores, asymmetries) needs further investigation. This would require intentional inclusion of youth of each of the BMI by age weight categories for both boys and girls. As performance on the FMS in youth is influenced by so many variables, a better understanding of the dynamic changes that occur in youth could be clarified by a large-scale study that includes a comparison of the FMS scores, asymmetries, and dysfunctional scores in inactive, physically active, and athletic boys and girls in different age groups. Although there are several training studies found in the literature, it appears that these studies evaluate the effectiveness of warm-up and training routines implemented for the purpose of research. Not present in the research is data collected after the cessation of the intervention. In other words, are the improvements gained during a training period maintained, or lost following several weeks or months after the program ends? Longitudinal studies that track changes in performance on the FMS over longer periods of time in physical education or athletic programs would also be beneficial. This would reveal the benefits of chronic participation for the participants and the program as whole.

## Data Availability

Not applicable.
